# Antimicrobial activity of antibiotic-induced resistant lactobacilli against carbapenemase-producing *Enterobacterales*

**DOI:** 10.3389/fmicb.2026.1931966

**Published:** 2026-10-22

**Authors:** Shao-Chuan Chen, Chi-Chung Chen, Chih-Cheng Lai, Hung-Jen Tang

**Affiliations:** 1 Department of Internal Medicine, Chi Mei Medical Center, Tainan, Taiwan; 2 Department of Medical Research, Chi Mei Medical Center, Tainan, Taiwan; 3 Department of Intensive Care Medicine, Chi Mei Medical Center, Tainan, Taiwan

**Keywords:** antibiotic-induced resistant, carbapenemase, *Enterobacterales*, *in vitro* activity, lactobacilli

## Abstract

**Background:**

Carbapenemase-producing *Enterobacterales* (CPE) are a critical global health threat with limited treatment options. Lactobacilli have been reported to exhibit antimicrobial activity against CPE, but whether this activity, together with key probiotic characteristics, is preserved after antibiotic-resistance induction—a clinically relevant condition given the concurrent antibiotic exposure that colonized or infected patients often receive—has not been characterized.

**Methods:**

Wild-type *Lactiplantibacillus plantarum* (LYC1322), *Lacticaseibacillus paracasei* (LYC1154), and *Lacticaseibacillus rhamnosus* (LYC1511), along with antibiotic-induced resistant variants generated by serial sublethal exposure to ampicillin, minocycline, and erythromycin (designated “ame”), were tested against 17 clinical CPE isolates spanning six species and five carbapenemase types (KPC, NDM, OXA-181, IMP-8, VIM-1) using an *in vitro* co-culture assay. Gastrointestinal tolerance (pepsin and oxgall assays) and Caco-2 epithelial adhesion were also evaluated for all six strains.

**Results:**

Both wild-type and ame-induced lactobacilli variants substantially reduced CPE viability, reducing recoverable CPE to below the limit of detection (<10 CFU/mL) for the majority of isolates by 24 h and for all 17 isolates by 48 h, compared with CPE-only controls sustaining 10^6^–10^9^ CFU/mL. This activity was consistent across all six species and all five carbapenemase types tested. Under simulated gastric (pepsin, pH 3.0) and intestinal bile salt (0.3% oxgall) conditions, ame-induced variants showed no obvious reduction in viability relative to their wild-type parental strains, although bile tolerance varied appreciably between strains; adhesion to Caco-2 monolayers appeared similar between wild-type and ame-induced variants at both inoculum densities tested.

**Conclusion:**

Under the *in vitro* conditions tested, antibiotic-resistance induction was not associated with an obvious reduction in anti-CPE activity, gastrointestinal stress tolerance, or epithelial adhesion, and the induced variants remained active against all CPE isolates examined, although bile tolerance differed between strains. Because reversibility testing and whole-genome sequencing were not performed, the induced phenotype may represent stable acquired rather than adaptive resistance, and the safety assessment of these variants remains incomplete. These findings are therefore preliminary and hypothesis-generating; mechanistic, genomic, animal, and clinical studies, including evaluation under concurrent antibiotic therapy—are required before any adjunctive probiotic application can be considered.

## 1 Introduction

Carbapenem-resistant *Enterobacterales* (CRE) have emerged as a major global public health threat and have been designated by the World Health Organization as a critical-priority pathogen requiring urgent attention (Sati et al., 2024). CRE infections are associated with substantial morbidity and mortality, particularly among critically ill patients, immunocompromised individuals, and solid organ transplant recipients (Martin et al., 2018; Paniagua-García et al., 2024). The widespread dissemination of carbapenemase-producing *Enterobacterales* (CPE), driven by enzymes such as *Klebsiella pneumoniae* carbapenemase (KPC), New Delhi metallo-β-lactamase (NDM), OXA-48-like carbapenemases, imipenemase (IMP), and Verona integron-encoded metallo-β-lactamase (VIM), has markedly limited available treatment options (Tamma et al., 2024; Hansen, 2021; Liu et al., 2026). Although agents such as colistin, tigecycline, and fosfomycin have traditionally served as salvage therapies, their clinical utility is increasingly challenged by rising rates of resistance. More recently developed β-lactam/β-lactamase inhibitor combinations, including ceftazidime-avibactam, remain active against many KPC- and OXA-48-producing isolates but have limited activity against metallo-β-lactamase-producing organisms (Tamma et al., 2024). Given these therapeutic limitations, additional treatment strategies are needed to improve outcomes in patients with CPE infections (Chao et al., 2026).

Lactobacilli, a group of lactic acid bacteria (LAB) that constitute an important component of the human microbiota, have attracted increasing attention as probiotic organisms because of their ability to modulate gut microbial communities and protect against enteric pathogens. Lactobacilli exert antimicrobial effects through multiple mechanisms, including the production of organic acids, bacteriocins, and hydrogen peroxide, as well as competitive exclusion of pathogens from epithelial adhesion sites (Shokryazdan et al., 2014; Chen et al., 2019; Chen et al., 2021; Tang et al., 2022). Recent studies have shown that certain lactobacilli can develop increased resistance to antibiotics following sublethal antibiotic exposure, a phenomenon referred to as antibiotic-induced resistance (Sulaiman and Lam, 2021). Such antibiotic-induced resistance has been attributed in some organisms to transcriptional and metabolic changes and in others to stable genetic alterations; these possibilities cannot be distinguished by susceptibility testing alone (Deventer et al., 2024). However, it remains unclear whether antibiotic-induced resistance enhances the ability of LAB to survive gastrointestinal stress while preserving their probiotic characteristics and antimicrobial activity.

Although previous investigations have reported inhibitory activity of wild-type lactobacilli against various multi-drug-resistant organisms including CRE (Shokryazdan et al., 2014; Chen et al., 2019; Chen et al., 2021; Tang et al., 2022), the antimicrobial potential of antibiotic-induced resistant LAB specifically against CPE has not been characterized. In patients with CPE colonization or infection who require concurrent antibiotic treatment, conventional probiotics are suppressed by the administered antibiotics, limiting their antagonistic effect. Whether lactobacilli that can survive antibiotic exposure retain their antimicrobial activity and probiotic properties has not been established. Therefore, we conducted this study to evaluate the *in vitro* antimicrobial activity of wild-type and antibiotic-induced resistant *Lactiplantibacillus plantarum* (LYC1322), *Lacticaseibacillus paracasei* (LYC1154), and *Lacticaseibacillus rhamnosus* (LYC1511), generated by induction with ampicillin, minocycline, and erythromycin, against various clinical CPE isolates representing five carbapenemase types (KPC, NDM, OXA-181, IMP-8, and VIM-1) across six species, and to assess the impact of resistance induction on their probiotic characteristics.

## 2 Methods

### 2.1 Bacterial strains and culture conditions

Three lactobacilli were used in this study: *Lacticaseibacillus paracasei* LYC1154, *Lactiplantibacillus plantarum* LYC1322, and *Lacticaseibacillus rhamnosus* LYC1511. All three strains, formerly assigned to the genus *Lactobacillus*, have been reclassified under the current *Lactobacillaceae* taxonomy (Zheng et al., 2020). Species confirmation was performed by 16S rDNA sequencing. A fragment of the 16S rDNA was amplified by polymerase chain reaction (PCR) as previously reported (Cruciani et al., 2015). All strains were maintained in de Man, Rogosa, and Sharpe (MRS; Oxoid Inc., Ogdensburg, NY, United States) broth (Oxoid, UK) at 37 °C conditions.

The CPE isolates comprised *Escherichia coli* (*n* = 5; carbapenemase types: VIM-1, IMP-8, KPC-2, OXA-181, NDM-5), *Klebsiella pneumoniae* (*n* = 5; KPC-2, OXA-181, NDM-1, VIM-1, IMP-8), *Citrobacter freundii* (n = 2; VIM-1, IMP-8), *Citrobacter koseri* (*n* = 1; OXA-181), *Enterobacter cloacae* (n = 3; NDM-1, VIM-1, IMP-8), and *Serratia marcescens* (*n* = 1; IMP-8). CPE isolates were collected from clinical specimens, including sputum, rectal swabs, blood, and urine. Species confirmations were performed using the VITEK 2 automated system (bioMérieux, Marcy l’Etoile, France) with standard biochemical methods. Mueller Hinton (MH) broth (Difco Laboratories, Detroit, MI, United States) was used for bacterial pathogens. The carbapenemase genes were confirmed by PCR and sequencing as described previously (Lai et al., 2016; Tang et al., 2019). In brief, DNA was extracted to be used as a template, and PCR was used to amplify target gene using specific primers (Lin et al., 2010). The study was approved by the Institutional Review Board (IRB) of Chi Mei Medical Center under approval number 11410–015.

### 2.2 Induction of antibiotic resistance in lactobacilli

Antibiotic resistance was induced in all three lactobacilli by serial sublethal exposure to three antibiotics: ampicillin, minocycline, and erythromycin. Briefly, overnight cultures were exposed to sub-inhibitory concentrations of each antibiotic over successive passages, with concentrations incrementally increased to select for reduced antibiotic susceptibility. The resulting antibiotic-induced resistant variants are designated with the suffix “ame” (LYC1154ame, LYC1322ame, LYC1511ame), reflecting the combined ampicillin–minocycline–erythromycin induction regimen. The rationale for selecting ampicillin, minocycline, and erythromycin for sequential induction was based on their distinct mechanisms of action, clinical relevance, and the stepwise findings of our induction experiments. Ampicillin, a beta-lactam, was initially selected as a representative cell wall synthesis inhibitor because of its widespread use in clinical and veterinary settings and was used to assess the initial capacity of the lactobacilli to develop reduced susceptibility. Following ampicillin induction, the minimum inhibitory concentrations (MICs) of most tested antibiotics increased in at least two of the three lactobacilli, with the exception of several agents, including minocycline and erythromycin. To further investigate the response to these relatively unaffected protein synthesis inhibitors, minocycline induction was subsequently performed. This resulted in a strain-specific increase in the MIC of minocycline in LYC1322, but not in LYC1154 or LYC1511. Finally, erythromycin induction was performed to evaluate macrolide-specific induction and resulted in a consistent increase in the erythromycin MIC across all three tested strains.

Lactobacilli were cultured in MRS broth at 37 °C. For resistance induction, each strain was exposed to the corresponding antibiotic at one-half of its minimum inhibitory concentration (0.5 × MIC). The antibiotic-containing broth was renewed every two days, and the MIC was re-determined weekly; whenever the MIC increased, the antibiotic concentration was reset to one-half of the newly determined MIC (stepwise escalation). Induction with each antibiotic was continued for a maximum of four consecutive weeks, whichever occurred first. Wild-type strains passaged in parallel under identical conditions without antibiotic served as passage-matched controls. The three antibiotics were applied sequentially: ampicillin-induced isolates were subsequently subjected to the identical minocycline induction protocol, and the resulting isolates were then exposed to erythromycin using the same step-up method (Hong et al., 2016).

### 2.3 Antimicrobial susceptibility testing by broth microdilution

MICs of 30 antimicrobial agents representing 11 drug classes were determined for all six lactobacilli variants (three wild-type and three ame-induced) using the broth microdilution method (Chen et al., 2019; Klare et al., 2005) with testing conditions referenced to CLSI M45 (3rd ed.) guidance for infrequently isolated or fastidious bacteria. Because species-specific clinical breakpoints are not established for lactobacilli, MIC values are reported without susceptible/intermediate/resistant categorization. Where available, the microbiological cut-off values defined by the European Food Safety Authority (EFSA) FEEDAP Panel and the tentative epidemiological cut-off values proposed for *Lactobacillaceae* species were used as a reference to distinguish wild-type from non-wild-type phenotypes (Rychen et al., 2018; Nøhr-Meldgaard et al., 2023). The agents tested were selected to span the major antibiotic classes relevant to clinical use, and validated interpretive cut-offs are available for only a subset; for the remaining agents, only MIC values are reported. Analyses focused on the fold-change in MIC between wild-type and induced variants rather than categorical susceptibility interpretation. Susceptibility testing was performed with two independent biological replicates (n = 2).

### 2.4 Gastrointestinal tolerance assays

#### 2.4.1 Pepsin tolerance assay

Pepsin tolerance was assessed to evaluate survival under simulated gastric conditions. Lactobacilli were adjusted to approximately 10^8^–10^9^ colony-forming units (CFU)/mL (exact 0 h counts are given in Table 1). Aliquots were incubated in simulated gastric fluid consisting of PBS adjusted to pH 3.0 containing pepsin (3 mg/mL; Sigma-Aldrich, St. Louis, MO, United States), as well as in a neutral pH 7.0 control (PBS only) at 37 °C under anaerobic conditions. Viable colony counts (CFU/mL) were determined by serial 10-fold dilution and plating on MRS agar at 0, 2, and 4 h of incubation. All assays were conducted with two independent biological replicates (*n* = 2) (Tokatlı et al., 2015). Acid tolerance was expressed as the log10 change in viable count relative to the 0 h inoculum [log10(Nt/N0)], which accounts for differences in the starting inoculum density among strains.

**Table 1:** Acid tolerance of wild-type and antibiotic-induced resistant (ame) lactobacilli under pepsin challenge (pH 3.0).

Strain	0 h inoculum (CFU/mL)	pH 3.0, 2 h (CFU/mL)	Survival 2 h (log10)	pH 3.0, 4 h (CFU/mL)	Survival 4 h (log10)	pH 7.0 control, 4 h (CFU/mL)
LYC1154	4.25 × 10^8^ ± 1.1 × 10^8^	6.00 × 10^7^ ± 7.1 × 10^6^	−0.85	6.25 × 10^7^ ± 1.1 × 10^7^	−0.83	3.25 × 10^8^ ± 3.5 × 10^7^
LYC1154ame	4.25 × 10^9^ ± 3.5 × 10^8^	4.50 × 10^8^ ± 7.1 × 10^7^	−0.98	2.25 × 10^8^ ± 3.5 × 10^7^	−1.28	1.25 × 10^9^ ± 2.1 × 10^8^
LYC1322	4.25 × 10^9^ ± 1.1 × 10^9^	6.25 × 10^8^ ± 1.1 × 10^8^	−0.83	5.75 × 10^8^ ± 2.5 × 10^8^	−0.87	1.65 × 10^9^ ± 3.5 × 10^8^
LYC1322ame	6.25 × 10^9^ ± 3.5 × 10^8^	3.50 × 10^8^ ± 7.1 × 10^7^	−1.25	2.40 × 10^8^ ± 7.1 × 10^6^	−1.42	1.53 × 10^9^ ± 1.8 × 10^8^
LYC1511	4.00 × 10^8^	5.25 × 10^8^ ± 1.8 × 10^8^	+0.12	7.50 × 10^7^	−0.73	9.75 × 10^8^ ± 2.5 × 10^8^
LYC1511ame	7.00 × 10^8^ ± 2.1 × 10^8^	5.50 × 10^8^ ± 1.4 × 10^8^	−0.10	4.25 × 10^8^ ± 3.5 × 10^7^	−0.22	1.28 × 10^9^ ± 1.8 × 10^8^

Viable counts are mean ± SD (CFU/mL) of duplicate experiments. Survival is expressed as the log10 change relative to the 0 h inoculum [log10(Nt/N0)]. In the pH 7.0 controls, counts remained at 10^8^–10^9^ CFU/mL throughout.

#### 2.4.2 Bile salt(Oxgall) tolerance assay

Bile salt tolerance was assessed using the oxgall assay. Lactobacilli were inoculated at a starting density of approximately 10^5^ CFU/mL (exact 0 h counts are given in Table 2) into MRS broth supplemented with 0.3% (w/v) oxgall (Sigma-Aldrich, St. Louis, MO, United States), which represents physiological bile salt concentrations in the small intestine. Unsupplemented MRS broth served as the growth control. Cultures were incubated at 37 °C under anaerobic conditions, and viable colony counts were determined by serial dilution and plating on MRS agar at 0, 4, and 24 h. Assays were performed with two independent biological replicates (*n* = 2) (Jacobsen et al., 1999). Bile tolerance was expressed as the percentage of the viable count in oxgall-supplemented broth relative to the unsupplemented (bile-free) control at the same time point.

**Table 2:** Bile tolerance of wild-type and antibiotic-induced resistant (ame) lactobacilli under 0.3% oxgall challenge.

Strain	0 h (CFU/mL)	oxgall, 4 h (CFU/mL)	% of control, 4 h	oxgall, 24 h (CFU/mL)	% of control, 24 h	control, 24 h (CFU/mL)
LYC1154	1.65 × 10^5^ ± 1.4 × 10^4^	2.08 × 10^5^ ± 3.9 × 10^4^	105%	5.50 × 10^8^ ± 2.1 × 10^8^	52%	1.05 × 10^9^ ± 2.8 × 10^8^
LYC1154ame	7.00 × 10^5^ ± 7.1 × 10^4^	7.25 × 10^5^ ± 1.1 × 10^5^	72%	6.50 × 10^8^ ± 1.4 × 10^8^	39%	1.68 × 10^9^ ± 5.3 × 10^8^
LYC1322	7.50 × 10^5^ ± 7.1 × 10^4^	1.98 × 10^6^ ± 3.2 × 10^5^	33%	7.00 × 10^7^ ± 2.1 × 10^7^	5%	1.40 × 10^9^
LYC1322ame	7.75 × 10^5^ ± 1.1 × 10^5^	5.25 × 10^5^ ± 3.5 × 10^4^	52%	6.75 × 10^8^ ± 1.1 × 10^8^	46%	1.48 × 10^9^ ± 3.9 × 10^8^
LYC1511	4.00 × 10^4^ ± 7.1 × 10^3^	5.25 × 10^5^ ± 1.1 × 10^5^	60%	1.93 × 10^9^ ± 2.5 × 10^8^	107%	1.80 × 10^9^ ± 1.4 × 10^8^
LYC1511ame	6.75 × 10^5^ ± 3.5 × 10^4^	7.00 × 10^5^ ± 1.4 × 10^5^	112%	2.38 × 10^8^ ± 1.1 × 10^7^	68%	3.50 × 10^8^ ± 1.4 × 10^8^

Viable counts are mean ± SD (CFU/mL) of duplicate experiments. Bile tolerance is expressed as the percentage of the viable count in oxgall relative to the bile-free control at the same time point.

### 2.5 *In vitro* co-culture assay against CPE

The activity of wild-type and antibiotic-induced resistant lactobacilli against seventeen CPE isolates was evaluated using an *in vitro* co-culture model. CPE isolates were adjusted to a concentration of 10^6^ CFU/mL in MRS broth, and lactobacilli were adjusted to 10^7^ CFU/mL, yielding a lactobacilli-to-CPE ratio of 10:1. Equal volumes of each suspension were mixed and co-incubated in tubes containing 10 mL of MRS-MH broth (1:1) at 37 °C under aerobic conditions to favor CPE growth while allowing lactobacilli viability (Chen et al., 2021; Drago et al., 1997). At 24 and 48 h, co-culture aliquots were serially diluted and plated on MacConkey agar (selective for *Enterobacterales*) to enumerate residual CPE colony counts. CPE-only controls (without lactobacilli) were included in parallel to confirm growth and establish baseline counts. The reduction in recoverable CPE was categorized by the log10 reduction relative to the corresponding CPE-only control at the same time point: 2–3 log10, 3–4 log10, and >4 log10 reduction (Figure 1). The limit of detection (LOD) for the traditional culture method (spread plate) is inherently restricted by the inoculation volume, typically yielding an LOD of 10 CFU/mL when plating 0.1 mL of sample (Lee et al., 2020). All experiments were conducted with two independent biological replicates (*n* = 2).

**Figure 1:**
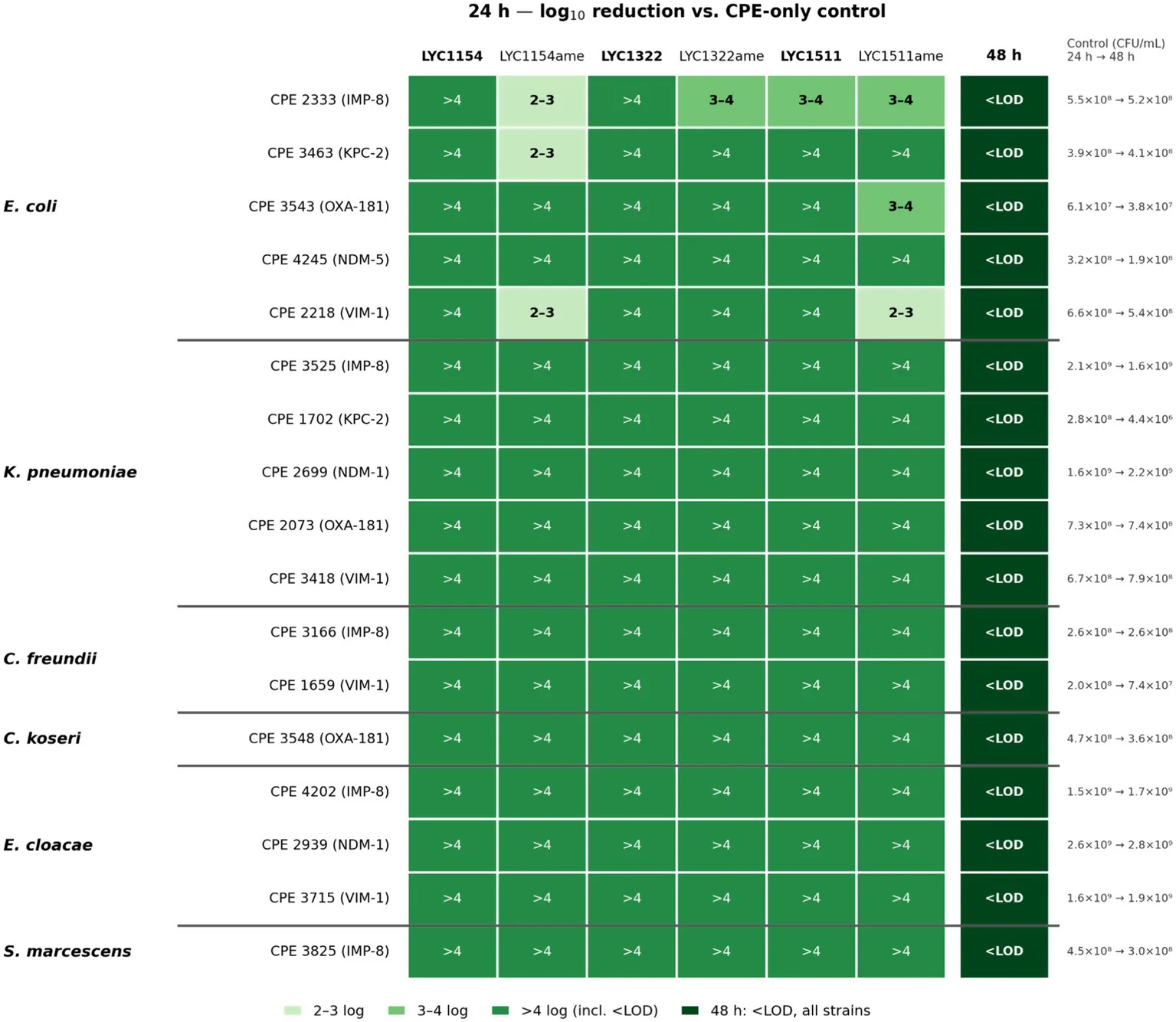
Reduction of recoverable carbapenemase-producing *Escherichia coli, Klebsiella pneumoniae, Citrobacter freundii, Citrobacter koseri, Enterobacter cloacae,* and *Serratia marcescens* clinical isolates after co-culture with lactobacilli. Each cell shows the log10 reduction in recoverable CPE relative to the corresponding CPE-only control at 24 h (color-graded: 2–3, 3–4, or >4 log10; darker indicates greater reduction, including counts below the limit of detection [LOD, <10 CFU/mL]). At 48 h, all six wild-type and antibiotic-induced resistant (ame) strains reduced every isolate to below the LOD (<10 CFU/mL). CPE-only controls remained at 10^6^–10^9^ CFU/mL over 48 h (values shown at right). Data are the mean of two independent biological replicates (*n* = 2); full viable counts (mean ± SD) are provided in Supplementary Table S1. Color grading and asterisk symbols denote log10-reduction categories relative to the corresponding CPE-only control and do not represent statistical significance; no inferential statistical testing was performed. WT, wild-type; ame, ampicillin–minocycline–erythromycin-induced variant.

### 2.6 Caco-2 cell adhesion assay

Adhesion of lactobacilli to intestinal epithelial cells was assessed using the human colorectal adenocarcinoma cell line Caco-2 (ATCC HTB-37). Caco-2 cells were seeded at 2 × 10^5^ cells/well in 24-well tissue culture plates and maintained in Dulbecco’s Modified Eagle’s Medium (DMEM) supplemented with 10% fetal bovine serum (FBS) and 1% penicillin–streptomycin at 37 °C in a 5% CO₂ humidified incubator for 48 h to allow monolayer formation.

Lactobacilli were prepared in antibiotic-free DMEM at concentrations of 10^8^ and 10^9^ CFU/mL. Medium was removed from Caco-2 monolayers, and 0.2 mL of each bacterial suspension was added to each well. Plates were incubated at 37 °C with 5% CO₂ for 2 h to allow adhesion. Non-adherent bacteria were removed by three washes with PBS. Cell-associated bacteria were recovered by adding 0.2% Triton X-100 (200 μL/well) to lyse the Caco-2 cells, followed by mechanical disruption using a cell scraper and repeated aspiration through a 1 mL syringe. The recovered suspension was serially diluted and plated on MRS agar for CFU enumeration. The multiplicity of infection (MOI) was defined as the ratio of lactobacilli CFU to Caco-2 cell number. All assays were performed with two independent biological replicates (n = 2) (Sharma and Kanwar, 2017).

### 2.7 Statistical analysis

Each assay was performed with two independent biological replicates (n = 2). A biological replicate was defined as a separate experiment initiated from an independent overnight culture on a different day, whereas technical replicates refer to repeated measurements within a single culture. Unless otherwise stated, quantitative data are expressed as the mean ± standard deviation (SD) of the two biological replicates. Because two biological replicates are insufficient to reliably estimate variance, no inferential statistical testing was performed, and comparisons between wild-type and antibiotic-induced resistant (ame) variants are descriptive. In the co-culture assay, the reduction in recoverable CPE was categorized by the log10 reduction relative to the corresponding CPE-only control: 2–3 log10 (*), 3–4 log10 (**), and >4 log10 (***); these symbols denote reduction categories, not statistical significance.

## 3 Results

### 3.1 Antibiotic susceptibility profiles of wild-type and antibiotic-induced resistant lactobacilli

The MIC profiles of wild-type and ame-induced lactobacilli variants are summarized in Table 3. Ampicillin MICs increased 8- to 16-fold in all three ame variants relative to their respective wild-type strains, with elevations of 8- to 16-fold in penicillin, 4- to 64-fold in amoxicillin-clavulanate, and 4- to 16-fold in ampicillin-sulbactam MICs. Among macrolides, erythromycin MICs rose to >128 μg/mL in LYC1154ame and LYC1511ame (from ≤0.125 μg/mL), and to 8 μg/mL in LYC1322ame; azithromycin MICs increased to a similar degree across all three induced variants. Carbapenem MICs were elevated after induction, although the magnitude varied by agent and strain. Doripenem MICs increased 8-fold in LYC1154ame (2 to 16 μg/mL) and LYC1322ame (≤0.125 to 1 μg/mL) and 4-fold in LYC1511ame (4 to 16 μg/mL); ertapenem MICs rose 4-fold in LYC1154ame (8 to 32 μg/mL) and LYC1511ame (16 to 64 μg/mL) and 16-fold in LYC1322ame (1 to 16 μg/mL); imipenem MICs increased 4- to 8-fold across the three strains (LYC1154, 2 to 8; LYC1322, ≤0.125 to 1; LYC1511, 2 to 16 μg/mL); and meropenem MICs increased 2-fold in LYC1154ame and LYC1511ame (4 to 8 μg/mL) and at least 32-fold in LYC1322ame (≤0.125 to 4 μg/mL). Clindamycin MICs increased from baseline values of 0.25–0.5 μg/mL to 8–16 μg/mL in all ame variants. For tetracyclines, minocycline MICs increased 8-fold in LYC1154ame (≤0.125 to 1 μg/mL) and 8-fold in LYC1322ame (2 to 16 μg/mL), whereas LYC1511ame showed no change. MICs of aztreonam (>128 μg/mL), colistin (>128 μg/mL), vancomycin (>128 μg/mL), teicoplanin (64 μg/mL), and fusidic acid (16–128 μg/mL, strain-dependent) remained unchanged or slight rise in all ame variants compared with their wild-type counterparts.

**Table 3:** Minimum inhibitory concentration (MIC) values of wild-type and antibiotic-induced resistant lactobacilli strains.

Lactobacilli strain	MIC value (mg/L)					
	LYC1154		LYC1322		LYC1511	
	Wild type	Ame-induced	Wild type	Ame-induced	Wild type	Ame-induced
Ampicillin	2	32	1	16	2	16
Penicillin	0.5	8	2	16	0.5	4
Amoxicillin-clavulanate	1/0.5	16/8	0.5/0.25	32/16	1/0.5	4/2
Ampicillin-sulbactam	4/2	64/32	4/2	16/8	4/2	16/8
Ceftazidime-avibactam	64/4	128/4	2/4	64/4	32/4	>128/4
Piperacillin-tazobactam	2/2	16/4	1/4	16/4	1/4	4/4
Ticarcillin-clavulanate	16/2	128/2	64/2	64/2	8/2	64/2
Cefoperazone-sulbactam	8/4	8/4	32/16	>128/64	8/4	64/32
Cefazolin	32	32	16	128	32	>128
Cefuroxime	8	64	2	32	8	64
Cefotaxime	16	16	0.25	2	8	>128
Cefepime	32	64	≤0.13	1	64	>128
Aztreonam	>128	>128	>128	>128	>128	>128
Doripenem	2	16	≤0.125	1	4	16
Ertapenem	8	32	1	16	16	64
Imipenem	2	8	≤0.125	1	2	16
Meropenem	4	8	≤0.125	4	4	8
Amikacin	64	128	32	32	16	16
Gentamicin	32	32	8	8	8	8
Kanamycin	>128	>128	128	128	64	64
Erythromycin	≤0.125	>128	≤0.125	8	≤0.125	>128
Azithromycin	0.5	>128	0.25	64	0.5	>128
Minocycline	≤0.125	1	2	16	≤0.125	≤0.125
Ciprofloxacin	4	8	64	64	4	8
Levofloxacin	2	4	8	8	4	4
Colistin	>128	>128	>128	>128	>128	>128
Trimethoprim-sulfamethoxazole	8	8	0.5	1	>128	>128
Clindamycin	0.25	16	0.5	16	0.25	8
Vancomycin	>128	>128	>128	>128	>128	>128
Teicoplanin	64	64	64	64	64	64
Fusidic acid	64	64	16	16	128	128

MIC values are expressed in mg/L (equivalent to μg/mL). Because species-specific clinical breakpoints are not established for lactobacilli, MICs are reported without susceptible/intermediate/resistant categorization.

### 3.2 Gastrointestinal tolerance

Because the starting inocula differed among strains (0 h counts ranged from 4.0 × 10^8^ to 6.3 × 10^9^ CFU/mL in the pepsin assay and from 4.0 × 10^4^ to 7.8 × 10^5^ CFU/mL in the oxgall assay), gastrointestinal tolerance was expressed as survival relative to the starting inoculum (pepsin) or relative to the bile-free control (oxgall) rather than as absolute counts.

Under pepsin challenge at pH 3.0, all six lactobacilli retained viability with less than 1.5 log10 reduction relative to their starting inocula over 4 h, and the ame variants appeared similar to their respective wild-type strains at both time points (Table 1). Bile tolerance, in contrast, varied appreciably between strains (Table 2). At 24 h, viable counts in 0.3% oxgall ranged from 5 to 107% of the corresponding bile-free control: LYC1511 was the most bile-tolerant wild-type strain (107% of control) and LYC1322 the least (5% of control), whereas the three ame variants fell within a narrower range (39–68% of control). The direction of change after induction was also strain-dependent, with LYC1322ame showing a higher percentage than its parental strain (46% vs. 5%), LYC1511ame a lower percentage than LYC1511 (68% vs. 107%), and LYC1154ame a value similar to that of LYC1154 (39% vs. 52%). Overall, no obvious reduction in gastric or bile tolerance was observed in the ame variants under the conditions tested, although these strain-specific differences are described without inferential testing given the duplicate design.

### 3.3 Reduction in recoverable carbapenemase-producing *Enterobacterales*

The reduction in recoverable CPE by all six lactobacilli against the 17 CPE isolates is presented in Table 4 and Figure 1; full viable counts (mean ± SD) at 24 and 48 h are provided in Supplementary Table S1.

**Table 4:** Reduction of recoverable carbapenemase-producing *Escherichia coli*, *Klebsiella pneumoniae*, *Citrobacter freundii*, *Enterobacter cloacae*, and *Serratia marcescens* clinical isolates after 24 h of co-culture.

Isolate (carbapenemase)	LYC1154	LYC1154ame	LYC1322	LYC1322ame	LYC1511	LYC1511ame	Control
*Escherichia coli*							
CPE 2333 (IMP-8)	2.80 × 10^3^*** ± 5.66 × 10^2^	4.80 × 10^6^* ± 2.83 × 10^5^	<10***	2.00 × 10^5^** ± 2.83 × 10^4^	4.40 × 10^5^** ± 8.49 × 10^4^	4.60 × 10^5^** ± 2.83 × 10^4^	5.50 × 10^8^ ± 9.90 × 10^7^
CPE 3463 (KPC-2)	<10***	4.30 × 10^5^* ± 9.90 × 10^4^	<10***	4.70 × 10^3^*** ± 2.40 × 10^3^	<10***	4.10 × 10^3^*** ± 2.97 × 10^3^	3.90 × 10^8^ ± 4.24 × 10^7^
CPE 3543 (OXA-181)	1.00 × 10^2^*** ± 1.41 × 10^2^	4.40 × 10^3^*** ± 1.41 × 10^3^	<10***	3.00 × 10^2^*** ± 1.41 × 10^2^	2.10 × 10^3^*** ± 1.27 × 10^3^	3.50 × 10^4^** ± 4.24 × 10^3^	6.10 × 10^7^ ± 1.27 × 10^7^
CPE 4245 (NDM-5)	<10***	<10***	<10***	1.00 × 10^2^*** ± 1.41 × 10^2^	<10***	<10***	3.20 × 10^8^ ± 2.83 × 10^7^
CPE 2218 (VIM-1)	1.00 × 10^2^*** ± 1.41 × 10^2^	5.30 × 10^6^* ± 1.56 × 10^6^	1.50 × 10^3^*** ± 9.90 × 10^2^	5.70 × 10^3^*** ± 2.12 × 10^3^	4.40 × 10^4^*** ± 1.13 × 10^4^	7.10 × 10^5^* ± 1.84 × 10^5^	6.60 × 10^8^ ± 5.66 × 10^7^
*Klebsiella pneumoniae*							
CPE 3525 (IMP-8)	<10***	8.00 × 10^2^*** ± 2.83 × 10^2^	<10***	8.00 × 10^2^*** ± 5.66 × 10^2^	4.70 × 10^3^*** ± 7.08 × 10^2^	<10***	2.10 × 10^9^ ± 4.24 × 10^8^
CPE 1702 (KPC-2)	<10***	<10***	<10***	<10***	<10***	<10***	2.80 × 10^8^ ± 2.83 × 10^7^
CPE 2699 (NDM-1)	<10***	<10***	<10***	<10***	<10***	<10***	1.60 × 10^9^ ± 2.83 × 10^8^
CPE 2073 (OXA-181)	<10***	<10***	<10***	<10***	<10***	<10***	7.30 × 10^8^ ± 1.27 × 10^8^
CPE 3418 (VIM-1)	4.10 × 10^3^*** ± 7.07 × 10^2^	<10***	<10***	<10***	<10***	<10***	6.70 × 10^8^ ± 4.24 × 10^7^
*Citrobacter freundii*							
CPE 3166 (IMP-8)	<10***	<10***	<10***	<10***	<10***	<10***	2.60 × 10^8^ ± 2.83 × 10^7^
CPE 1659 (VIM-1)	<10***	<10***	<10***	<10***	<10***	<10***	2.00 × 10^8^ ± 2.83 × 10^7^
*Citrobacter koseri*							
CPE 3548 (OXA-181)	<10***	<10***	<10***	<10***	<10***	<10***	4.70 × 10^8^ ± 7.07 × 10^7^
*Enterobacter cloacae*							
CPE 4202 (IMP-8)	<10***	<10***	<10***	<10***	<10***	<10***	1.50 × 10^9^ ± 1.41 × 10^8^
CPE 2939 (NDM-1)	<10***	<10***	<10***	<10***	<10***	<10***	2.60 × 10^9^ ± 5.66 × 10^8^
CPE 3715 (VIM-1)	<10***	<10***	<10***	<10***	<10***	<10***	1.60 × 10^9^ ± 0.00 × 10^0^
*Serratia marcescens*							
CPE 3825 (IMP-8)	<10***	<10***	<10***	<10***	<10***	<10***	4.50 × 10^8^ ± 1.41 × 10^7^

Reduction in recoverable CPE is expressed as the log10 reduction relative to the corresponding CPE-only control: *, 2–3 log10 reduction; **, 3–4 log10 reduction; ***, >4 log10 reduction. These asterisks denote reduction categories only and do not indicate statistical significance. Data are mean ± SD (CFU/mL) from two independent biological replicates (*n* = 2), <10, below the limit of detection (<10 CFU/mL). Control, CPE-only culture without lactobacilli.

All six wild-type and antibiotic-induced resistant (ame) lactobacilli reduced every isolate to below the limit of detection (<10 CFU/mL) at 48 h, whereas CPE-only controls remained at 10^6^–10^9^ CFU/mL; therefore only 24-h data are shown.

At 24 h, co-culture of carbapenemase-producing *E. coli* with wild-type lactobacilli produced greater than 4 log10 reductions for most isolates, with counts below the limit of detection (<10 CFU/mL) for CPE 3463 (KPC-2) and CPE 4245 (NDM-5). The ame-induced strains reduced recoverable *E. coli* by 2–3 to greater than 4 log10 at 24 h, depending on strain and isolate (Figure 1). By 48 h, all six strains, both wild-type and ame-induced, had reduced every *E. coli* isolate to below the limit of detection.

Against all five *K. pneumoniae* isolates (KPC-2, OXA-181, NDM-1, VIM-1, and IMP-8), the majority of co-cultures reduced counts to below the limit of detection at 24 h, and all six strains reduced every isolate to below the limit of detection by 48 h.

Against both *C. freundii* isolates (CPE 3166, IMP-8; CPE 1659, VIM-1) and the single *C. koseri* isolate (CPE 3548, OXA-181), all six lactobacilli reduced recoverable counts to below the limit of detection (<10 CFU/mL) at both 24 and 48 h, whereas the corresponding CPE-only controls remained at 7.4 × 10^7^–4.7 × 10^8^ CFU/mL over both time points.

Similarly, against all three *E. cloacae* isolates (CPE 4202, IMP-8; CPE 2939, NDM-1; CPE 3715, VIM-1) and the single *S. marcescens* isolate (CPE 3825, IMP-8), all six strains, both wild-type and ame-induced, reduced recoverable counts to below the limit of detection (<10 CFU/mL) at both time points. The corresponding CPE-only controls remained high throughout, at 1.5 × 10^9^–2.8 × 10^9^ CFU/mL for *E. cloacae* and at 4.5 × 10^8^ (24 h) and 3.0 × 10^8^ (48 h) CFU/mL for *S. marcescens*.

### 3.4 Cell adhesion to Caco-2 cells

Adhesion indices for all six lactobacilli variants at both inoculum concentrations (10^8^ and 10^9^ CFU/mL) are presented in Figure 2. Both wild-type and ame-induced strains adhered to Caco-2 monolayers at both inoculum concentrations, and adhesion by the ame-induced variants appeared similar to that of their wild-type counterparts, with no obvious reduction under the conditions tested.

**Figure 2:**
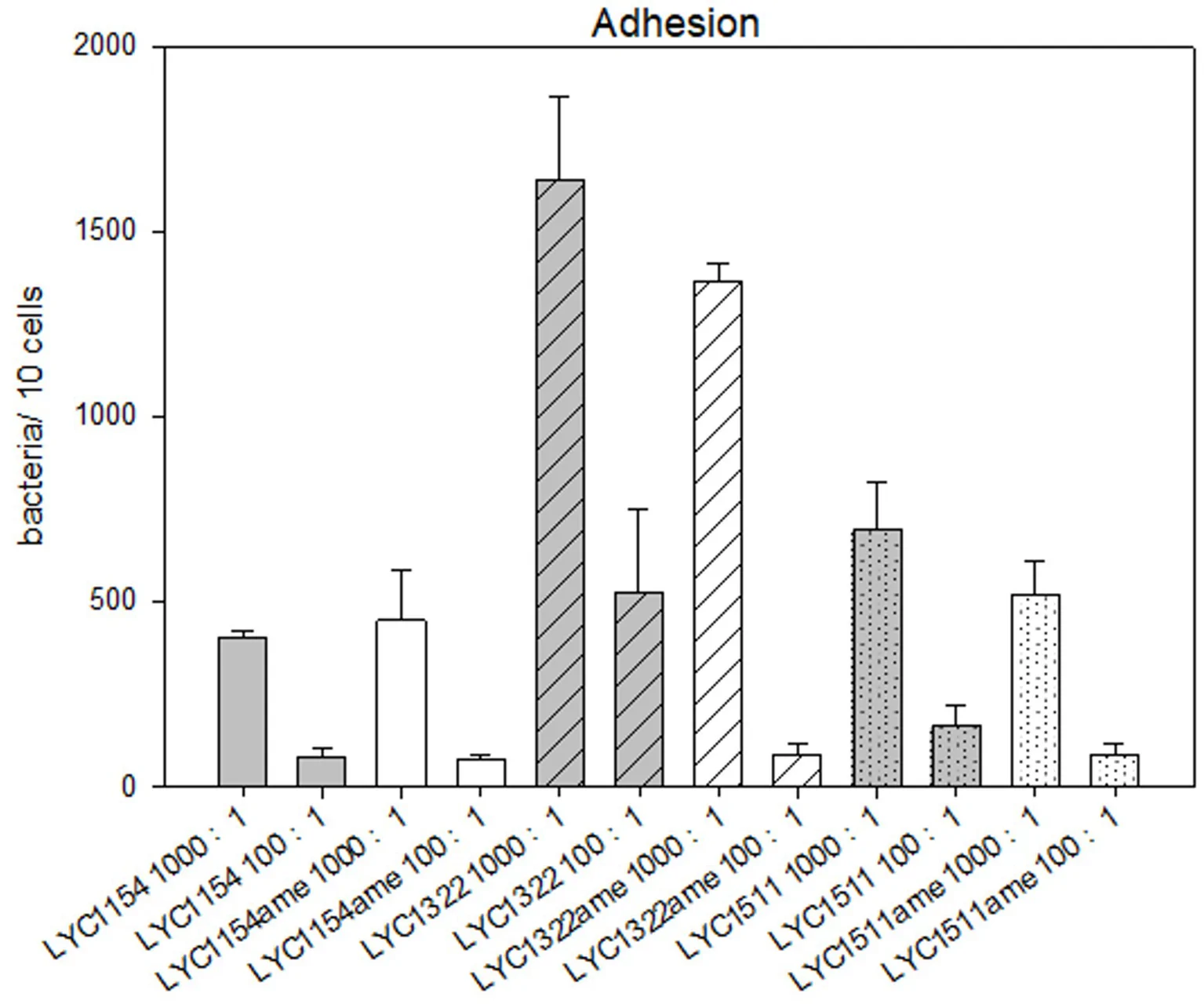
Cell adhesion of wild-type LYC1154, LYC1322, and LYC1511 and their ame-induced variants to Caco-2 monolayers at two multiplicities of infection (1,000:1 and 100:1). Data are the mean ± SD of two independent biological replicates (*n* = 2); comparisons between wild-type and ame variants are descriptive.

## 4 Discussion

This study demonstrated that antibiotic resistance induced in *Lcb. paracasei* LYC1154, *Lpb. plantarum* LYC1322, and *Lcb. rhamnosus* LYC1511 through serial sublethal exposure to ampicillin, minocycline, and erythromycin was not associated with an obvious loss of activity against CPE. The antibiotic-induced resistant variants remained active against clinical isolates spanning six species and all five major carbapenemase types (KPC-2, NDM, OXA-181, IMP-8, and VIM-1), and showed pepsin tolerance, bile-salt tolerance, and Caco-2 epithelial adhesion generally consistent with those of their wild-type parental strains. Bile tolerance, however, differed appreciably between strains, both among the wild-type strains themselves and in the direction of change after induction; characteristics of this kind are strain-specific properties, and any candidate strain taken forward would require individual characterization rather than extrapolation from the group as a whole. To our knowledge, this is the first study to characterize both the antimicrobial and probiotic properties of antibiotic-induced resistant lactobacilli specifically against CPE.

These findings build on previous studies, including our own, showing that wild-type lactobacilli inhibit CRE through organic acid and bacteriocin production, hydrogen peroxide generation, and competitive exclusion from epithelial adhesion sites (Chen et al., 2019; Chen et al., 2021). The anti-CRE effects of lactobacilli have been observed across different species and experimental models. A vaginal *Limosilactobacillus fermentum* isolate reduced mortality in murine carbapenem-resistant *Klebsiella* infection (Tajdozian et al., 2021), while *Lpb. plantarum* PMC105 provided complete protection against systemic CRE infection in a neutropenic and dysbiotic mouse model (Sarafraz et al., 2025). While these studies focused on wild-type strains, our results show that anti-CPE activity was still observed after resistance induction, suggesting that induced resistance does not necessarily abolish antimicrobial function under the conditions tested.

The anti-CPE activity of these strains has been mechanistically characterized in our earlier work rather than in the present study. In that work, activity was abolished by neutralizing cell-free supernatants to pH 6.5, while catalase and proteinase K treatment had no effect, identifying lactic acid as the principal effector (Chen et al., 2021). Activity was still observed after resistance induction, but this observation does not by itself indicate that the same molecular mechanism is operating in the induced variants; preservation of activity does not necessarily imply preservation of the underlying mechanism. Antibiotic-induced resistance in lactobacilli may be accompanied by metabolic and transcriptional changes that could alter organic acid production or introduce additional effectors. The effector responsible for activity in the ame variants was not identified here, and confirming whether the acid-mediated mechanism is preserved would require cell-free supernatant neutralization, catalase, and proteinase K experiments performed on these variants; we therefore draw no mechanistic conclusions from the present data.

The potential clinical implications of these findings lie in the setting in which probiotics would be used. Patients colonized or infected with CPE often receive concurrent antibiotic treatment, and probiotics must be able to survive and function in an antibiotic-rich environment to be effective. This challenge was highlighted in a randomized placebo-controlled trial in which an eight-strain probiotic mixture was no more effective than placebo in eradicating extended-spectrum β-lactamase-producing *Enterobacterales*, partly because the administered probiotics may have been eliminated by concurrent antibiotic therapy (Ljungquist et al., 2020). Antibiotic-induced resistant lactobacilli may, in principle, help overcome this limitation. Among current microbiome-based approaches for CPE control, such as fecal microbiota transplantation and short-chain fatty acid supplementation (Zhang et al., 2025), probiotics capable of surviving antibiotic exposure may represent a practical adjunctive strategy to reduce intestinal CPE colonization, an important reservoir for subsequent infection and hospital transmission (Tajdozian et al., 2021; Sarafraz et al., 2025).

The induced phenotype is a form of antibiotic resistance rather than tolerance or persistence, both of which involve prolonged survival at bactericidal concentrations without a rise in MIC (Brauner et al., 2016; Balaban et al., 2019). Resistance itself spans a spectrum: adaptive resistance is a transient, often reversible rise in MIC driven by physiological or transcriptional changes under continued exposure, whereas stable acquired resistance reflects heritable genetic change persisting after antibiotic withdrawal. As the genetic basis of the large MIC increases observed here was not determined, we describe the variants as antibiotic-induced resistant without yet distinguishing adaptive from stable acquired resistance; reversibility testing and whole-genome sequencing are required to make this distinction. The MIC increases observed here were substantial, exceeding 1,000-fold for erythromycin and azithromycin in two of the three strains and reaching 8- to 64-fold for several beta-lactams and for clindamycin. A phenotype of this magnitude may represent stable acquired resistance and should not be assumed to be transient or adaptive; because neither reversibility testing after antibiotic withdrawal nor genomic characterization was performed, the present data cannot distinguish between these possibilities.

Because the induced phenotype is a form of resistance, its safety implications require careful consideration. The decisive criterion is not reduced susceptibility itself but whether it is transferable: genus-wide genomic data indicate that acquired resistance genes flanked by mobile genetic elements are uncommon in lactobacilli and largely restricted to species other than those studied here (Campedelli et al., 2019), and long-term ampicillin exposure in *Lpb. plantarum* has been associated predominantly with protein-homeostasis changes rather than mobile genetic elements (Cao et al., 2020). Nonetheless, prolonged sublethal exposure is itself a recognized risk, having been reported to induce penicillin G resistance in some lactobacilli (Walter et al., 2019) and to facilitate β-lactamase gene exchange among *Enterobacterales* (Kunishima et al., 2019). The safety of these induced variants therefore remains unestablished in the present study. Demonstrating the absence of transferable resistance determinants by whole-genome sequencing to exclude acquired and mobilizable resistance genes, by transfer or conjugation assays, and by testing phenotypic stability after antibiotic withdrawal is an essential prerequisite rather than an optional addition, and must precede any further therapeutic development. Until such data are available, the present findings should be regarded as a microbiological proof of concept and not as support for clinical use.

This study had several limitations. First, all experiments were conducted *in vitro* and may not fully reflect conditions in the human gastrointestinal tract, where interactions with resident microbiota, mucus, host immunity, and other environmental factors may influence probiotic activity. The findings therefore require confirmation in animal models and, ultimately, clinical studies. Second, the co-culture assays used a fixed lactobacilli-to-CPE ratio of 10:1, did not include concurrent antibiotic exposure—so the hypothesis that the induced variants would remain active during concurrent antibiotic therapy remains untested—and did not measure pH; moreover, the agents used to induce resistance do not necessarily correspond to those used clinically to treat CPE, and whether similar activity occurs under other conditions remains to be determined. Third, although antibiotic resistance was confirmed phenotypically by serial increases in MIC, the molecular mechanisms underlying the observed resistance were not investigated. In particular, whole-genome sequencing, screening for acquired resistance genes, and analysis of genetic changes are needed to determine whether the increased resistance resulted from physiological adaptation or stable genetic alterations. The long-term stability of the resistance phenotype also remains unknown. In addition, the induced variants were not re-confirmed by molecular species identification, and a single induced lineage was generated for each strain, so reproducibility across independent lineages remains to be established. Further molecular characterization—including whole-genome sequencing, transferability testing, and assessment of phenotypic stability—together with *in vivo* studies will therefore be necessary before any therapeutic application can be considered. Fourth, although the CPE panel included all five major carbapenemase types, the number of isolates representing each species and carbapenemase type was limited, which may affect the generalizability of the findings. Finally, each assay was performed with only two independent biological replicates (n = 2); because this number is insufficient to support robust inferential statistics, the data are reported descriptively, which limits the strength of the between-strain comparisons.

In conclusion, this study demonstrated that serial exposure to sublethal concentrations of ampicillin, minocycline, and erythromycin generated antibiotic-induced resistant lactobacilli variants that showed no obvious reduction in anti-CPE activity or in the probiotic characteristics examined. These strains remained active against multiple *Enterobacterales* species carrying the major carbapenemase types, with *in vitro* gastrointestinal stress tolerance and epithelial adhesion generally consistent with those of their parental strains, although bile tolerance was strain-dependent. These findings provide preliminary *in vitro* evidence that antibiotic-induced resistant lactobacilli may warrant further investigation as a potential approach for managing CPE colonization or infection in the setting of concurrent antibiotic exposure, but in the absence of animal and clinical validation they remain *in vitro* observations only. Importantly, the safety assessment of these variants is incomplete: the genetic basis and transferability of the resistance phenotype were not determined, and deliberate administration of antibiotic-resistant organisms to patients cannot be contemplated until transferable resistance determinants have been formally excluded. Further mechanistic, genomic, and *in vivo* investigations are therefore needed to determine the safety, efficacy, and potential clinical applicability of these strains.

## Data Availability

The original contributions presented in the study are included in the article/Supplementary material, further inquiries can be directed to the corresponding authors.
