# Supplementary material for: Antimicrobial activity of antibiotic-induced resistant lactobacilli against carbapenemase-producing Enterobacterales
Source: Front Microbiol. 2026 Oct 22;17:1931966. doi: 10.3389/fmicb.2026.1931966 (PMC13736769; doi:10.3389/fmicb.2026.1931966)
Supplement: Supplementary file 1 [file Data_Sheet_1.DOCX]

**Supplementary Table S1.** Reduction of recoverable carbapenemase-producing *Escherichia coli*, *Klebsiella pneumoniae*, *Citrobacter freundii*, *Citrobacter koseri*, *Enterobacter cloacae*, and *Serratia marcescens* clinical isolates after 24 and 48 h of co-culture with wild-type and antibiotic-resistance-induced (ame) lactobacilli. Values are viable counts (CFU/mL) expressed as mean ± SD of duplicate experiments; <10 denotes counts below the limit of detection (10 CFU/mL). Symbols denote the log10 reduction relative to the corresponding CPE-only control at 24 h: *, 2–3 log10; **, 3–4 log10; ***, >4 log10.

| **Isolate (carbapenemase)** | **Strain** | **24 h CFU/mL (mean ± SD)** | **48 h CFU/mL (mean ± SD)** |
| --- | --- | --- | --- |
| ***Escherichia coli*** | | | |
| **CPE 2333 (IMP-8)** | LYC1154 | 2.80×10³ ± 5.66×10² *** | <10 |
|  | LYC1154ame | 4.80×10⁶ ± 2.83×10⁵ * | <10 |
|  | LYC1322 | <10 | <10 |
|  | LYC1322ame | 2.00×10⁵ ± 2.83×10⁴ ** | <10 |
|  | LYC1511 | 4.40×10⁵ ± 8.49×10⁴ ** | <10 |
|  | LYC1511ame | 4.60×10⁵ ± 2.83×10⁴ ** | <10 |
|  | *CPE-only control* | 5.50×10⁸ ± 9.90×10⁷ | 5.20×10⁸ ± 8.49×10⁷ |
| **CPE 3463 (KPC-2)** | LYC1154 | <10 | <10 |
|  | LYC1154ame | 4.30×10⁵ ± 9.90×10⁴ * | <10 |
|  | LYC1322 | <10 | <10 |
|  | LYC1322ame | 4.70×10³ ± 2.40×10³ *** | <10 |
|  | LYC1511 | <10 | <10 |
|  | LYC1511ame | 4.10×10³ ± 2.97×10³ *** | <10 |
|  | *CPE-only control* | 3.90×10⁸ ± 4.24×10⁷ | 4.10×10⁸ ± 4.24×10⁷ |
| **CPE 3543 (OXA-181)** | LYC1154 | 1.00×10² ± 1.41×10² *** | <10 |
|  | LYC1154ame | 4.40×10³ ± 1.41×10³ *** | <10 |
|  | LYC1322 | <10 | <10 |
|  | LYC1322ame | 3.00×10² ± 1.41×10² *** | <10 |
|  | LYC1511 | 2.10×10³ ± 1.27×10³ *** | <10 |
|  | LYC1511ame | 3.50×10⁴ ± 4.24×10³ ** | <10 |
|  | *CPE-only control* | 6.10×10⁷ ± 1.27×10⁷ | 3.80×10⁷ ± 5.66×10⁶ |
| **CPE 4245 (NDM-5)** | LYC1154 | <10 | <10 |
|  | LYC1154ame | <10 | <10 |
|  | LYC1322 | <10 | <10 |
|  | LYC1322ame | 1.00×10² ± 1.41×10² *** | <10 |
|  | LYC1511 | <10 | <10 |
|  | LYC1511ame | <10 | <10 |
|  | *CPE-only control* | 3.20×10⁸ ± 2.83×10⁷ | 1.90×10⁸ ± 9.90×10⁷ |
| **CPE 2218 (VIM-1)** | LYC1154 | 1.00×10² ± 1.41×10² *** | <10 |
|  | LYC1154ame | 5.30×10⁶ ± 1.56×10⁶ * | <10 |
|  | LYC1322 | 1.50×10³ ± 9.90×10² *** | <10 |
|  | LYC1322ame | 5.70×10³ ± 2.12×10³ *** | <10 |
|  | LYC1511 | 4.40×10⁴ ± 1.13×10⁴ *** | <10 |
|  | LYC1511ame | 7.10×10⁵ ± 1.84×10⁵ * | <10 |
|  | *CPE-only control* | 6.60×10⁸ ± 5.66×10⁷ | 5.40×10⁸ ± 5.66×10⁷ |
| ***Klebsiella pneumoniae*** | | | |
| **CPE 3525 (IMP-8)** | LYC1154 | <10 | <10 |
|  | LYC1154ame | 8.00×10² ± 2.83×10² *** | <10 |
|  | LYC1322 | <10 | <10 |
|  | LYC1322ame | 8.00×10² ± 5.66×10² *** | <10 |
|  | LYC1511 | 4.70×10³ ± 7.08×10² *** | <10 |
|  | LYC1511ame | <10 | <10 |
|  | *CPE-only control* | 2.10×10⁹ ± 4.24×10⁸ | 1.60×10⁹ ± 5.66×10⁸ |
| **CPE 1702 (KPC-2)** | LYC1154 | <10 | <10 |
|  | LYC1154ame | <10 | <10 |
|  | LYC1322 | <10 | <10 |
|  | LYC1322ame | <10 | <10 |
|  | LYC1511 | <10 | <10 |
|  | LYC1511ame | <10 | <10 |
|  | *CPE-only control* | 2.80×10⁸ ± 2.83×10⁷ | 4.40×10⁶ ± 8.49×10⁵ |
| **CPE 2699 (NDM-1)** | LYC1154 | <10 | <10 |
|  | LYC1154ame | <10 | <10 |
|  | LYC1322 | <10 | <10 |
|  | LYC1322ame | <10 | <10 |
|  | LYC1511 | <10 | <10 |
|  | LYC1511ame | <10 | <10 |
|  | *CPE-only control* | 1.60×10⁹ ± 2.83×10⁸ | 2.20×10⁹ ± 5.66×10⁸ |
| **CPE 2073 (OXA-181)** | LYC1154 | <10 | <10 |
|  | LYC1154ame | <10 | <10 |
|  | LYC1322 | <10 | <10 |
|  | LYC1322ame | <10 | <10 |
|  | LYC1511 | <10 | <10 |
|  | LYC1511ame | <10 | <10 |
|  | *CPE-only control* | 7.30×10⁸ ± 1.27×10⁸ | 7.40×10⁸ ± 5.66×10⁷ |
| **CPE 3418 (VIM-1)** | LYC1154 | 4.10×10³ ± 7.07×10² *** | <10 |
|  | LYC1154ame | <10 | <10 |
|  | LYC1322 | <10 | <10 |
|  | LYC1322ame | <10 | <10 |
|  | LYC1511 | <10 | <10 |
|  | LYC1511ame | <10 | <10 |
|  | *CPE-only control* | 6.70×10⁸ ± 4.24×10⁷ | 7.90×10⁸ ± 1.56×10⁸ |
| ***Citrobacter freundii*** | | | |
| **CPE 3166 (IMP-8)** | LYC1154 | <10 | <10 |
|  | LYC1154ame | <10 | <10 |
|  | LYC1322 | <10 | <10 |
|  | LYC1322ame | <10 | <10 |
|  | LYC1511 | <10 | <10 |
|  | LYC1511ame | <10 | <10 |
|  | *CPE-only control* | 2.60×10⁸ ± 2.83×10⁷ | 2.60×10⁸ ± 1.41×10⁸ |
| **CPE 1659 (VIM-1)** | LYC1154 | <10 | <10 |
|  | LYC1154ame | <10 | <10 |
|  | LYC1322 | <10 | <10 |
|  | LYC1322ame | <10 | <10 |
|  | LYC1511 | <10 | <10 |
|  | LYC1511ame | <10 | <10 |
|  | *CPE-only control* | 2.00×10⁸ ± 2.83×10⁷ | 7.40×10⁷ ± 0 |
| ***Citrobacter koseri*** | | | |
| **CPE 3548 (OXA-181)** | LYC1154 | <10 | <10 |
|  | LYC1154ame | <10 | <10 |
|  | LYC1322 | <10 | <10 |
|  | LYC1322ame | <10 | <10 |
|  | LYC1511 | <10 | <10 |
|  | LYC1511ame | <10 | <10 |
|  | *CPE-only control* | 4.70×10⁸ ± 7.07×10⁷ | 3.60×10⁸ ± 2.83×10⁷ |
| ***Enterobacter cloacae*** | | | |
| **CPE 4202 (IMP-8)** | LYC1154 | <10 | <10 |
|  | LYC1154ame | <10 | <10 |
|  | LYC1322 | <10 | <10 |
|  | LYC1322ame | <10 | <10 |
|  | LYC1511 | <10 | <10 |
|  | LYC1511ame | <10 | <10 |
|  | *CPE-only control* | 1.50×10⁹ ± 1.41×10⁸ | 1.70×10⁹ ± 1.41×10⁸ |
| **CPE 2939 (NDM-1)** | LYC1154 | <10 | <10 |
|  | LYC1154ame | <10 | <10 |
|  | LYC1322 | <10 | <10 |
|  | LYC1322ame | <10 | <10 |
|  | LYC1511 | <10 | <10 |
|  | LYC1511ame | <10 | <10 |
|  | *CPE-only control* | 2.60×10⁹ ± 5.66×10⁸ | 2.80×10⁹ ± 2.83×10⁸ |
| **CPE 3715 (VIM-1)** | LYC1154 | <10 | <10 |
|  | LYC1154ame | <10 | <10 |
|  | LYC1322 | <10 | <10 |
|  | LYC1322ame | <10 | <10 |
|  | LYC1511 | <10 | <10 |
|  | LYC1511ame | <10 | <10 |
|  | *CPE-only control* | 1.60×10⁹ ± 0 | 1.90×10⁹ ± 1.41×10⁸ |
| ***Serratia marcescens*** | | | |
| **CPE 3825 (IMP-8)** | LYC1154 | <10 | <10 |
|  | LYC1154ame | <10 | <10 |
|  | LYC1322 | <10 | <10 |
|  | LYC1322ame | <10 | <10 |
|  | LYC1511 | <10 | <10 |
|  | LYC1511ame | <10 | <10 |
|  | *CPE-only control* | 4.50×10⁸ ± 1.41×10⁷ | 3.00×10⁸ ± 5.66×10⁷ |
